# Ignoring the planet: A critical blind spot for research on ageing

**DOI:** 10.1111/joim.70032

**Published:** 2025-10-22

**Authors:** Paul Shiels, Ognian Neytchev, Gillian Borland, Polina Germushkina, Richard Johnson, Peter Stenvinkel, Tina Woods

**Affiliations:** ^1^ Glasgow Geroscience Group School of Molecular Biosciences MVLS University of Glasgow Glasgow UK; ^2^ The Department of Medicine University of Colorado Anschutz Medical Campus Aurora Colorado USA; ^3^ Department of Clinical Science Intervention and Technology Division of Renal Medicine Karolinska Institutet Huddinge Sweden; ^4^ Collider Health London UK

**Keywords:** ageing, epigenetics, exposome, geroscience, planetary health

## Abstract

Although research on ageing has largely concentrated on understanding the fundamental biology of the ageing process and devising pharmaceutical interventions in order to slow it down, increasing evidence has underscored the crucial role of environmental inputs across the life course and across generations, in shaping both individual and intergenerational trajectories of age‐related health. These include nutrition, air pollution, social deprivation, lifestyle factors, climate change and exposure to environmental toxins, including microplastics and nanoplastics. The development of the concept of the exposome of ageing and the emergence of the new field of ‘exposomics’ have identified a blind spot, in particular, for geroscience. The impact of the exposome affecting human ‘healthspan’ (i.e., years lived in good health), extending across generations, is significant and yet under‐explored in research. As such, it is under‐appreciated that the declining health of the planet will have intergenerational ripple effects, epigenetically priming adverse health in future generations. We discuss the capacity to manipulate our exposome to mitigate against such effects, by addressing root causes, rather than symptoms, of both physiological and planetary dysregulation, dysfunction and decay. We propose a systems‐based framework that reconnects research on ageing with exposomics and planetary ecology, creating a new field of ‘ecological or exposome pharmacology’, harnessing the activity of Nrf2 as a senotherapeutic intervention to improve trans‐ and intergenerational physiology in the face of declining planetary health.

## Graphical abstract

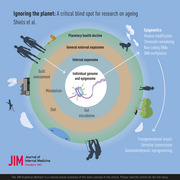

## Introduction

*There are in fact two things, science and opinion; the former begets knowledge, the latter ignorance. (Hippocrates of Kos 460‐377 BC)*

Two areas of contemporary research that have been generating extensive opinion are research on ageing and planetary health. There is a wide variety of public and professional opinions in these areas, which often leads to the scientific evidence being overlooked. Today, there are more people aged 60 years or older, than at any time in human history—a remarkable human achievement. Globally, in 2020, the number of people aged 60 years and older outnumbered children under 5 years. Between 2015 and 2050, the proportion of the world's population aged 60 years and older is expected to nearly double, from 12% to 22% (∼2.1 billion people), whereas the number of people aged 80 years and older is expected to triple (∼426 million) [1, 2].

**Fig. 1:**
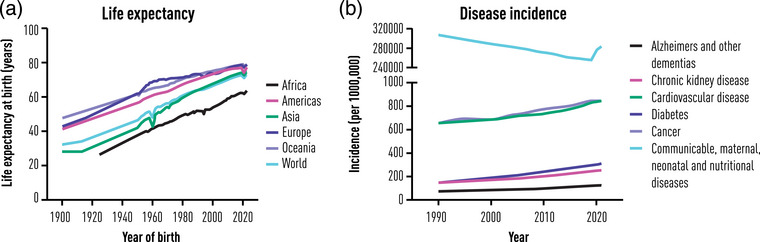
Regional global life expectancy and global disease incidence trends. (a) A rise in life expectancy is associated with (b) a worldwide increase in age‐related diseases. Source: (a) Data retrieved 9 September 2025 from https://archive.ourworldindata.org/20250909‐093708/grapher/life‐expectancy.html (archived on 9 September 2025); (b) Institute for Health Metrics and Evaluation (IHME), GBD Compare, Seattle, WA: IHME, University of Washington, 2015. Available from http://vizhub.healthdata.org/gbd‐compare. (Accessed 9/9/25).

Over the last 150 years, improvements in public health and medicine have resulted in a dramatic improvement in lifespan, so that today the global life expectancy at birth is 73.3 years, an increase of 8.4 years since 1995 [3]. However, this increase in lifespan has not been matched by an increase in healthspan. Consequently, the last decade of life is often spent living with a range of morbidities that negatively impact quality of life. This has resulted in the emergence of a ‘diseasome of ageing’ [4, 5], where distinct clinical disease modalities are all underpinned by a common component of a dysregulated ageing process [6, 7] (Fig. 1). Strikingly, we now know that the ageing process is malleable and that its progression can be slowed at the organismal level. Claims that it can be reversed at this level remain science fiction, though this remains a serious aim of research by longevity scientists. As a consequence, the geroscience hypothesis has evolved to posit that tackling upstream effectors of ageing will pre‐empt the onset of the ‘diseasome of ageing’ and compress morbidity, thus increasing healthspan and closing the gap with lifespan [8, 9, 10].

We have growing evidence, however, that age‐related healthspan is directly linked to bio‐psychosocial factors, in particular, socioeconomic position [11]. Addressing socioeconomic inequalities in health is thus crucial to global efforts to reduce healthcare costs in the areas of the world where the demographic of the aged is growing most, and where it is economically least affordable.

In this respect, the annual economic return of improving health in older age is multiple times greater than the cost of health interventions themselves. Healthcare costs decrease as the onset of chronic disease is delayed. Unsurprisingly, labour force participation and productivity increase if people stay healthier for longer. Overall, educational and economic outcomes improve across the life course with better health. McKinsey has estimated the cost of poor health in the United States at $2.3 trillion a year, and the disadvantaged suffer most. Conversely, improving health (both lifespan and healthspan) could add $12 trillion to global GDP by 2040—an 8% boost, driven largely by productivity gains and labour force participation [12].

A recent study of 40 countries has revealed that healthy (i.e., normative) and accelerated ageing are influenced by physical, social and sociopolitical exposomes, with considerable disparities across nations [13]. Europe led in healthy ageing, whereas Egypt and South Africa showed the greatest acceleration of ageing; Asia and Latin America were positioned between these, whereas accelerated ageing was more evident in eastern and southern Europe; globally, it was also associated with lower income [13]. Understanding the relationship between our planetary exposome and how we age is thus of increasing urgency, as at present we appear to be sleepwalking through this crisis.

## The ageing process

*There is no conclusion except age and decay. (Mandeville‘s Travels 14^th^ century)*

To better gauge the potential for any malleability in the process of ageing and to determine causality for any exposome factors affecting this process, one must be able to understand ageing at a fundamental biological level. Ageing can be described as a segmental and non‐linear, time‐dependent physiological decline, resulting from an accumulation of macromolecular deficits. It exhibits significant inter‐individual variation, with a high degree of intra‐individual variation between different organ systems (i.e., segmental ageing), resulting in an increase in age‐specific mortality and a decrease in age‐specific reproductive rates. Notably, ageing starts pre‐conception and ends at death [14]. It is not simply a collection of diseases in the final decades of life, nor is it a disease per se, as has been widely argued within clinical medicine. What is pertinent in this respect is that the pre‐conception phase of the ageing process is driven by trans‐ and intergenerational epigenetic factors in response to differing environmental exposures [15].

### Hallmarks of ageing

At present, ageing can be hallmarked into a series of features that are common across taxa. These comprise a series of 12 hallmarks of ageing that can be sub‐classified into primary, antagonistic and integrative (or secondary) hallmarks, based on their functional roles in the ageing process. These form the basis for targeted senotherapeutic interventions to ameliorate physiological and physical decline [16].

The primary hallmarks comprise the initial causes of macromolecular cellular damage and include genomic instability, telomere attrition, epigenetic dysregulation and loss of proteostasis. Antagonistic hallmarks function initially as protective mechanisms but become damaging when excessive or uncontrolled. These encompass dysregulated nutrient sensing, mitochondrial dysfunction, impaired autophagy and cellular senescence. Integrative (or secondary) hallmarks are those arising from primary and antagonistic damage and contribute to the final manifestations of the ageing process. These include stem cell exhaustion, altered intercellular communication and inflammageing. An additional hallmark is microbial dysbiosis, presently considered a systemic modifier of ageing and inflammageing [16].

The interventions targeting these hallmarks represent a broad spectrum of senotherapies. These are rapidly growing in number and are listed in breadth in Table 1. As can be seen from the range of agents in Table 1, research on ageing remains firmly embedded within a pharmacological model, where there remains a clear ambition to compress morbidity and delay age‐related physical and physiological decline through pharmacological intervention. However, this approach treats ageing with an emphasis on ‘sick care’ and not healthcare. Consequently, the use of senotherapies largely revolves around biomedical paradigms to develop pharmaceutical interventions to slow ageing, or with the ultimate long‐term aspiration of reversing ageing by manipulating the underlying molecular and cellular biology. This is a halcyon time for research on ageing, as it offers real promise to close the gap between healthspan and lifespan. However, despite this momentum, we still lack a robust scientific model to provide a holistic picture of how we age as humans. Despite years of research using animal models, most findings have not yet translated into tangible health outcomes for humans.

**Table 1:** Interventions in ageing.

Hallmark of ageing	Intervention class	Examples (pharmacological)	Examples (lifestyle/non‐pharma)
Genomic instability	DNA repair enhancers, gene therapy	NAD+ precursors, PARP modulators, CRISPR tools	Radiation avoidance, antioxidant‐rich diet
Telomere attrition	Telomerase activators	TA‐65, astragaloside IV, hTERT gene therapy	Meditation (stress reduction), physical activity
Epigenetic dysregulation	Epigenetic reprogramming	Yamanaka factors (OSKM), HDAC inhibitors	Caloric restriction, low‐methionine diet
Loss of proteostasis	Proteostasis/Autophagy enhancers	Rapamycin, spermidine, trehalose	Fasting, exercise
Deregulated nutrient sensing	Caloric restriction mimetics	Metformin, rapamycin, resveratrol, NAD+ boosters	Fasting, caloric restriction
Mitochondrial dysfunction	Mitochondria‐targeted therapies	MitoQ, SS‐31 (elamipretide), NAD+ precursors, CoQ10	Aerobic exercise
Cellular senescence	Senolytics/Senomorphics	Dasatinib + quercetin, fisetin, navitoclax	Senostatic diets, intermittent fasting
Stem cell exhaustion	Stem cell rejuvenation/therapy	GDF11, MSC or HSC transplantation	Exercise, blood donation (mild stimulation)
Altered intercellular communication	Anti‐inflammatory, immune modulation	Rapamycin, JAK inhibitors	Mediterranean diet, sleep optimization
Inflammageing	Anti‐inflammatory agents	Omega‐3s, JAK inhibitors, rapamycin anti‐IL6 inhibitors	CR, plant‐based diet, stress reduction
Disabled autophagy	Autophagy inducers	Rapamycin, spermidine, trehalose	Fasting, exercise
Dysbiosis	Microbiome modulators	Probiotics, prebiotics, faecal transplant (FMT)	High‐fibre diet, fermented foods

*Note*: Table listing examples of different classes of pharmacological and non‐pharmacological interventions targeting hallmarks of ageing.

We postulate that current research on ageing often under‐emphasizes the profound role of environmental exposures, especially the ‘foodome’ and thus the state of planetary health, in shaping not only individual health outcomes but transgenerational transmission of environmental effects [17, 18]. The prevalent reductionist approach to research on ageing, based within a standard pharmacological model, struggles to explain the complexity of inter‐individual variation in how we age and to account for transgenerational effects [19]. In particular, recent evidence suggests that the exposome, reflecting biotic and abiotic exposures—inclusive of broader ecological and lifestyle factors—may have greater long‐term impact, particularly when viewed through the lens of transgenerational health, not accounted for by standard models.

There is now a growing body of evidence indicating that as much as 80% of the trajectory of ageing and health is driven by the exposome and not genetics [20]. It is therefore obvious that any changes in the exposome will impact human health outcomes and the underlying ageing process.

Prior to the development of the exposome concept [21], the role of environmental conditions in shaping the process of ageing—in particular, social environmental conditions—was first demonstrated in UK cohorts in Glasgow, which demonstrated that cumulative social deprivation was biologically embedded via inflammatory, epigenetic and microbiome‐related pathways that accelerated ageing and resulted in earlier onset of age‐related disease in deprived communities [11, 22, 23, 24, 25, 26, 27]. More recently, this was further developed at superior power by the LIFEPATH (Horizon‐2020) consortium. LIFEPATH prioritized life‐course exposures and applied the exposome concept, using multi‐omics platforms in harmonized European cohorts to again identify biological pathways linking social disadvantage to accelerated ageing and disease risk [28, 29, 30]. The role of epigenetics in linking life‐course environmental effects with dysregulated ageing was again prominent and for the first time demonstrated using contemporary epigenetic clocks [28].

### Epigenetics and ageing

**Fig. 2:**
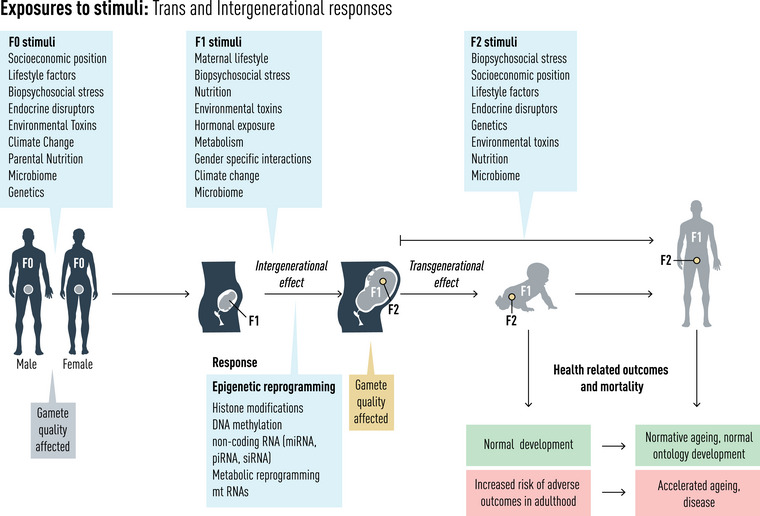
Exposures to stimuli: trans‐ and intergenerational responses. Illustration depicting how a range of exposome stressors can induce inter‐ and transgenerational epigenetic responses leading to aberrant development, accelerated ageing and adverse health outcomes in adult life.

Epigenetics refers to inheritable modifications in gene activity that occurs without altering the DNA sequence itself; essentially, changes in how genes are expressed without change to the genetic code. Epigenetic mechanisms support biological adaptability to environmental perturbations and can pass these adaptive responses to future generations. This is in keeping with epigenetic intergenerational responses (IGRs) and transgenerational responses (TGRs) observed in humans and other mammals [31, 32]. Epigenetic inheritance can thus be broadly categorized into these two types: IGRs and TGRs. Intergenerational effects arise when environmental influences, such as those encountered during prenatal development, affect the offspring directly, leading to changes that span one generation. In contrast, transgenerational inheritance occurs when epigenetic changes are passed down to subsequent generations that were not directly exposed to the original environmental factor that initiated the modification (Fig. 2).

These effects can be transmitted along both the maternal and paternal lines. Intergenerational effects typically result from maternal exposure (e.g., nutritional stress and toxins) and paternal exposures (stress and diet). Traditionally, it was thought that these effects were only maternally inherited, as mitochondrial DNA (mtDNA) is inherited exclusively along maternal lines, and sperm‐derived mtDNA is eliminated before fertilization [33]. However, it has since been discovered that sperm‐derived mtRNA can respond to environmental cues and affect subsequent offspring development and health trajectories [34, 35, 36, 37]. In both mice and humans, sperm‐derived mtRNAs have now been demonstrated to mediate the effects of paternal diet. For example, in mice whose fathers consumed high‐fat foods and in humans with fathers who had a high body mass index, their male offspring developed metabolic disorders [38]. The mechanisms underpinning transcription of these sperm mtRNAs, and how they affect development in response to environmental cues, remain to be fully explored, but critically, they indicate causality for exposome factors driving this aspect of the ageing process (Fig. 2).

Increasing evidence indicates that epigenetic information can also be inherited transgenerationally across a range of species, including humans, with effects manifesting in the F2 generation (i.e., in grandchildren) not subject to the original exposure. In mice, both paternal stress and maternal stress exposures have resulted in transgenerational effects [18, 31]. Human data showing epigenetic inheritance transgenerationally have only been observational to date [32]. Two key epidemiological studies in humans provide the bulk of this information—namely, the Dutch famine birth cohort study and the Överkalix study from Sweden. The Dutch data show transgenerational transmission of effects from peri‐conceptional exposure—including increased neonatal adiposity, Type‐2 diabetes, schizophrenia and cardiovascular disease—often associated with hypomethylation of the *IGF2* gene, which contributes to insulin resistance [39, 40, 41, 42, 43].

The Överkalix study has observed that a paternal grandfather's pre‐pubertal food access predicts his male, but not female, grandchildren's all‐cause mortality. Cardiovascular mortality, however, was only associated with parental nutrition, not grandparental. More recent analyses, using a 40 times larger data set, have indicated that cancer mortality could be explained by TGR, where the nutritional environment of a paternal grandfather during his pre‐pubertal years was linked to cancer mortality in his grandsons. However, previous results for diabetes and cardiovascular mortality were not reproduced [44].

One consequence of TGR is that heritable ‘epimutations’ may contribute to heritable phenotypic variation and thus impact human evolution. The epigenetic landscape is dynamic in response to environmental changes and mediated by changes in genomic DNA methylation, ncRNAs and chromatin modification (generally histone modifications). These are also key features of the ageing process. The implications of the transmission of epigenetic effects between generations are profound. They imply that today's environmental exposures will affect the ageing trajectories of subsequent generations. Moreover, early life exposures may set the stage for dysregulation of the ageing processes decades before clinical symptoms emerge. Epigenetic drift—that is, age‐associated changes in DNA methylation—may itself be accelerated, or exacerbated, by these inherited environmental exposures. Essentially, healthspan is not only a matter of an individual's exposome, but their ancestral exposomes.

From this perspective, we contend that the current narrow biomedical focus on research on ageing—in particular, pharmacological‐based interventions driven by the geroscience hypothesis—has created a critical blind spot: the failure to recognize the exposome and fully integrate environmental determinants of health, especially those related to the degradation of planetary health. This domain, although often treated as peripheral, is in fact central to the fundamental biology of ageing, particularly when improved healthspan is viewed not just as an individual goal, but as a shared intergenerational and societal imperative, with profound economic impacts. This realization should reframe the focus of research on ageing, not as an isolated biomedical goal, but as an intergenerational responsibility and shared societal imperative.

## Broadening the lens: Linking ageing, food and biomimetic solutions

*It is better to treat the patient with food than with medicine… …the art of healing comes from nature, not from the physician. (Galen of Pergamum 129–216 AD)*

To address this blind spot, we propose a systems‐based framework that reconnects research on ageing with exposomics [45] and the ecological realities of planetary health. This is very much in keeping with the planetary health framework, first introduced and formalized by The Lancet Commission in 2015 [46], which acknowledged that human health and the health of Earth's natural systems are intertwined. Our approach sees declining planetary health as a proximate driver of ageing and disease. In this model, the environment is not merely a backdrop to human biology; it is a co‐determinant of health outcomes.

### The planetary connection: ecological degradation as a heritable health crisis

The Anthropocene has been defined as the geological period during which human activity has been the dominant influence on the climate and the environment. During this period, human activities have impacted the environment sufficiently to engender distinct planetary change. The Anthropocene has been characterized by climate instability, loss of biodiversity, air and water pollution, deforestation, soil degradation and habitat loss, industrial monocultural farming, a shortage of potable water, plus a zoonotic pandemic layered upon an ongoing sixth mass extinction and a growing aged human population demographic. The increasing ubiquity of persistent toxins—including organic pollutants, microplastics and industrial byproducts—means that exposures in utero may dysregulate the epigenome in ways that may not manifest for decades [4, 42, 44, 45]. Exposure to endocrine‐disrupting chemicals (EDCs)—such as DES (diethylstilbestrol) and BPA (bisphenol A)—has been documented to induce reproductive abnormalities, alter cancer risk profiles and cause behavioural alterations across multiple generations [47, 48]. This constitutes not only a planetary crisis, but also a crisis of biological inheritance and legacy for humanity.

Likewise, the anthropocenic foodome is engendering a nutritional collapse, tied to industrial agriculture, lower micronutrient density, diminished dietary diversity and reliance on ultra‐processed foods, all of which can adversely affect health [49, 50]. The industrialized microbiome, urbanization, antibiotic overuse and reduced contact with soil and animals may further dysregulate immune development with adverse biological and physiological consequences. The metabolic activity of soil microbiomes has a central role in global nutrient cycles [51].

Transgenerational epigenetics already provides a compelling framework for understanding how environmental factors, both salutogenic and pathogenic, shape the ageing process across time. This understanding reframes our understanding of the ‘ageing human’, not simply as an isolated organism, but also as a living archive of ancestral exposures and exposome interactions. It also underscores the moral and scientific imperative to protect both environmental and biological futures. To extend human longevity and improve healthspans without safeguarding the ecosystems that sustain us is not only short‐sighted, it is simply unsustainable and unachievable.

## Solutions: manipulating the exposome to improve physiological resilience

*Tell me what you eat and I will tell you what you are. (Jean Anthelme Brillat‐Savarin, 1755–1826)*

How might we align the needs of planetary health and human health, amidst the backdrop of an ageing demographic and related adverse health sequelae? One immediate and achievable strategic goal might be to improve physiological resilience in the face of climate change. This, of course, is intimately tied to planetary health. Ancestral and traditional food systems have typically developed in step with local ecologies, optimized for both health and sustainability. However, during the Anthropocene, industrialized food production and the emergence and propagation of the Western diet have dramatically changed this landscape. Modern food preservation techniques, while extending shelf‐life and reducing waste, have negatively impacted both human and planetary health, due to energy‐intensive processing contributing to greenhouse gas emissions, food system inefficiencies and unsustainable consumption patterns, all of which drive the ‘diseasome of ageing’ [52].

The Western diet, in particular, is abundant in ultra‐processed sugar‐rich foods that can directly contribute to the predisposition and progression of the metabolic disease [53, 54]. Additionally, the innate constituents and their production (i.e., the food and water used in their production) may contain xenobiotics, gerontogens, cytotoxins, phosphate additives, microplastics and nanoplastics [55, 56]. The ‘foodome’ represents the entire chemical composition of a food sample and/or how these interact with a biological system at a given time. The foodome thus displays a plethora of interactions with human biology at a mechanistic level. This includes modulating the epigenetic landscape [18, 57, 58], modulating the transcriptome and acting as a source of metabolic substrates used by the microbiome and, in turn, shaping the composition of that microbial community [50]. Additionally, the foodome is both directly and indirectly (via the microbiome) responsible for the regulation of cytoprotective responses mediated by nuclear factor erythroid 2–related factor 2 (Nrf2).

Nrf2 is a transcription factor that controls the expression of over 200 genes involved in antioxidant defence, xenobiotic metabolism, inflammation regulation and mitochondrial biogenesis [59]. By serving as a master regulator of cellular stress responses, Nrf2 acts as a molecular linchpin for enhancing physiological resilience in the face of both internal and external exposome stressors. Under conditions of oxidative or electrophilic stress, Nrf2 is released from its cytoplasmic inhibitor Keap1 and translocates to the nucleus, where it binds to antioxidant repeat elements (AREs) of a host of cytoprotective genes to initiate their transcription [60]. These genes then induce cytoprotective responses. Critically, Nrf2 activity declines with age, leading to a reduced capacity to respond to exposome stressors [61]. As a result, exposome stressors—such as heat stress, microplastics and air pollution (e.g., PM_2.5_, ozone and diesel exhaust)—exacerbate the chronic inflammation, impair mitochondrial function and alter the epigenetic landscape of ageing [62]. Additionally, food insecurity and poor diet quality associated with industrial agricultural and biodiversity loss reduce access to Nrf2‐activating phytochemicals (e.g., sulforaphane, curcumin and quercetin).

In support of a thesis arguing for activation of Nrf2 to enhance physiological resilience, biomimetic studies—that is, solutions found in the natural world that have evolved via natural selection to allow animals to adapt to exposome stresses—support this approach [53, 63, 64]. These indicate that superior antioxidant defence mechanisms with enhanced Nrf2 expression have evolved to protect animals living under extreme environmental conditions [65]. Hibernating species employ elevated Nrf2 levels to combat physiological stressors. Hibernation is a survival strategy that has evolved to deal with seasonal exposome stress—for example, food shortage, cold and oxidative stress. These animals include the hibernating ground squirrel (*Ictidomys tridecemlineatus*) and hibernating bats (*Myotis ricketti*), which undergo repeated ischaemia–reperfusion cycles, leading to high basal levels of ROS and RNS [66, 67].

Similarly, seals (Phocidae) subjected to extreme apnoea‐induced hyperoxaemia when diving and reperfusion stress when returning rapidly to the surface resist tissue injury by upregulating Nrf2 expression. Additionally, naked mole rats (*Heterocephalus glaber*) also cope with extreme environmental hypoxia via superior Nrf2 expression [68].

In humans, the Nrf2–Keap1 signalling pathway becomes naturally repressed with increasing age, a feature exacerbated in progeroid (accelerated ageing) syndromes, such as Hutchinson–Gilford progeria syndrome (HGPS) and reflected within the ‘diseasome of ageing’. In HGPS, caused by a mutant form of Lamin A termed progerin, the mutant protein traps Nrf2 at the nuclear periphery, driving an accelerated ageing phenotype. Exogenous reactivation of Nrf2 reverses this progerin‐induced cellular ageing [69]. Senotherapeutic activation of Nrf2—by nutrients or synthetic or semi‐synthetic Nrf2 activators and agonists—is thus a serious geroscientific strategy.

Additionally, the gut microbiome plays a critical role in activating Nrf2 pathways, which are central to cytoprotective responses [70]. Activation of Nrf2 can thus be achieved indirectly via a modulation of the microbiome. Salutogenic (i.e., health‐promoting) bacteria can process dietary‐derived polyphenols to produce alkyl catechols that activate Nrf2. These bacteria thrive on traditional diets incorporating fermentation for preservation [71], but not on the Western diet. Consequently, the natural microbial exposome has been significantly disrupted during the Anthropocene, which has seen the emergence of an industrialized microbiome [72]. A significant reduction in dietary fibre intake and reduced consumption of fermented foods in modern populations has led to microbial dysbiosis [73, 74]. In parallel with the emergence of the anthropocenic foodome and industrialised agriculture, a decline in soil and crop biodiversity has occurred alongside the evolution of the diseasome of ageing, resulting in microbial‐driven inflammation as a significant factor in the diseasome of ageing [75, 76, 77, 78, 79].

The Industrialized microbiome has continually evolved during the Anthropocene and is very different from that of our ancestors. Our ancestral microbiome evolved from a foraging, or hunter–gatherer lifestyle, that incorporated eating large amounts of plant material, including microbiota‐accessible carbohydrates (MACs). As a result, human metabolism adapted to having commensal fibrolytic microorganisms. The human metabolism has through evolution adapted to having commensal fibrolytic microorganisms and their metabolic products, such as short‐chain fatty acids (SCFAs) [80]. The advent and use of antibiotics have exacerbated the broad presentation of dysbiosis in the industrialized microbiota. Significantly, hunter–gatherer and subsistence farming populations possess a more diverse gut microbiome than their industrialized counterparts and retain a more normative array of ancestral microbial types [81]. It is pertinent to note that the microbiome is also susceptible to exposome perturbations brought on by heat stress and air pollution. Changes in temperature have been linked, in multiple phyla, to changes in microbial diversity and function [82, 83]. Air pollution has also been reported to adversely affect microbiome composition and diversity [84]. Microplastics can also negatively impact gut health by disrupting the gut microbiome [85]. Emerging research suggests that biodiversity loss also affects the human microbiome and, in turn, the gut–brain‐immunology axis, where Nrf2 plays a regulatory role [86]. The decline of microbial diversity in urban and post‐industrial populations has been linked to immune dysregulation [87, 88, 89, 90].

Supporting gut health by improving air quality, enhancing nutrition, reducing microplastic exposure and promoting adequate hydration can protect humans against exposome stressors. This offers another potential route for nutraceutical senotherapies. Although nearly 2000 plants are known sources of dietary fibre, only a few are widely used. These underutilized sources hold potential to modulate ageing‐related epigenetic processes in real time through bioactive compounds. This includes the adoption of epigenetic diets (i.e., a diet composed of foods and nutrients that can influence the epigenome or the processes underpinning the epigenetic landscape) or supplementation with betaine [19, 57].

Betaine (also known as tri‐methyl glycine), a common constituent of beets, is a methyl donor nutrient that feeds into the methionine cycle to preserve maintenance of the epigenome. Endogenous betaine production is lost as a consequence of mitochondrial dysregulation with ageing [73]. This naturally occurring compound is an osmolyte that has recently been reported to act as a geroprotectant, whereby exercise‐driven betaine enrichment rescued age‐related physiological deterioration in mice, by binding to and inhibiting TANK‐binding kinase 1 (TBK1) [91]. This is in keeping with observations in humans, where diminished betaine levels have been reported to be correlated with accelerated ageing and an imbalanced diet, low in nutritionally derived polyphenols [73]. Betaine supplementation extends lifespan and healthspan in a number of model organisms from differing taxa and in human cells in vitro (Tran and Shiels personal communication, 3/8/2025).

Additionally, biomimetic interventions—such as restoring a more traditional/normative microbiome (akin to rewilding the human microbiome), increasing exposure to salutogenic green spaces in urban settings or designing biophilic environments—may help mitigate against the adverse health consequences imposed by environmental stressors.

Urban environments can serve as valuable real‐world testbeds for understanding the human exposome and its impact on health, particularly for measuring the interaction of external and internal exposome factors, such as with the microbiome. Cities, with their inherent complexity and variability, offer a realistic and dynamic setting for applying exposomics methodologies. These approaches enable the systematic capture, measurement and analysis of the interactions between the external environment and an individual's internal biological state, thereby deepening our understanding of complex health outcomes beyond genetics [45]. This can be seen in the ‘real‐world’ societal testbeds for Doughnut Economics Action Lab projects in Glasgow, Barcelona, Tomelilla and Mexico City. These are designed to ameliorate adverse urban bio‐psychosocial exposome features for the respective general popualtions and to help mitigate adverse features of the geophysical exposome, including climate change, biodiversity and ocean acidification.

The salutogenic benefits of designing more biophilic environments, using biomimetic principles to improve the health of endogenous human populations, seem sensible in the face of planetary change [92, 93]. Examples include the Sahara Forest Project and the Eastgate Centre in Harare, with environments operating as a zero‐waste system and suitable to a wide variety of climates, which have been designed to reduce exposome stress and thus improve healthspan. Similarly, the Bosco Verticale in Milan—which features an urban forest integrated into a tower block—exemplifies effective biophilic design.

## Conclusions

What will be the epigenetic legacy of the Anthropocene? Environmental exposures experienced today may epigenetically prime intergenerational ripple effects leading to accelerated ageing and adverse health in future generations. Thus, as planetary systems falter, we may be encoding in our biology the very consequences of ecological imbalance. We propose that any consideration of healthspan must now extend beyond individual lifetimes. Senotherapeutic interventions must therefore address not only the needs of an ageing global population, but also ageing ecologies. As such, the integration of a geroscience approach to improve age‐related physiology across generations must integrate with an understanding of our planetary exposome. Coupled with protection of biodiversity, restoration of ecosystems and reduction of exposure to environmental toxins, these are not just acts of conservation, but acts of intergenerational healthcare.

Directly, or indirectly, harnessing the activity of Nrf2 offers a powerful tool for such senotherapeutic intervention. Crucially, it also bridges the gap between personalized approaches to improving healthspan and planetary health imperatives. This has the potential to create a new field of ‘ecological or exposome pharmacology’, targeting Nrf2 and other senotherapeutic strategies and harnessing exposomics as a tool to understand the complexities of the interplay between the exposome and health [45].

This would enable a systems‐based resilience strategy, offering protection against the growing exposome stress that defines the Anthropocene. Instead of addressing these symptoms in isolation, this approach would foster the development of endogenous cytoprotectants, such as Nrf2, through ecologically informed nutrition and habitat stewardship. Such a strategy may prove to be one of the most effective ways to enhance healthspan and longevity across individuals, populations and generations. As such, the pursuit of improved longevity and healthspan would extend beyond individual pursuits and become collective and intergenerational, with planetary goals benefiting everyone.

## Conflict of Interest statement

P. S. is funded by Revalesio (USA) and act as a Scientific Reviewer for Mars Petcare UK, sits on the Strategic Advisory Board of the Danone Institute and the Scientific Advisory Board of AKL Therapeutics. T. W. is Founder and CEO of Collider Health and Executive Director for the International Institute of Longevity. P. S. is a consultant or advises for Baxter, Astra Zeneca, GSK, Vifor, Alexion, CSL, Vera; Research Funding: Bayer, Astra Zeneca. He has received honoraria from Astra Zeneca, Baxter, Astellas, Novo Nordisk, Pfizer/BSM, FMC, CSL and has Advisory or Leadership Roles for Astra Zeneca, GSK, Alexion, CSL, Boehringer and Vafeso. R. J. is on the Scientific Board of Santa Barbara Nutrients, RxSugar, and a new dimension has equity with Colorado Research Partners LLC and consults with Kibow, Amgen and Sobi Pharmaceuticals.
